# A Rare Case Report of Periorbital Pseudolymphoma

**DOI:** 10.7759/cureus.36270

**Published:** 2023-03-16

**Authors:** Uroosa Subhan, Najia Ahmed, Tariq M Malik, Syed Arbab Shah, Unaiza Hasan

**Affiliations:** 1 Dermatology, PNS Shifa Hospital, Karachi, PAK; 2 Dermatology, Bahria University of Health Sciences, Karachi, PAK

**Keywords:** ace inhibitors, angioedema, triamcinolone acetonide, orbital disease, reactive lymphoid hyperplasia, pseudolymphoma

## Abstract

Pseudolymphoma (PSL) of the orbit is a benign lymphoid hyperplasia (LH). It is a rare disease with an extensive range of known causative agents. LH is further classified into "reactive" (RLH) and "atypical" (ALH) types. It clinically presents as a single or a few plaques and/or nodular lesions, particularly on the head, neck, and upper trunk. It must be differentiated from orbital malignant lymphoma. In this report, we present a case of a 58-year-old Pakistani female with an asymptomatic recurrent right periorbital swelling for three years. It was clinically diagnosed as an angiotensin-converting enzyme (ACE) inhibitor-induced angioedema as it responded to stopping the ACE inhibitor; however, after four months, the patient again started to develop right periorbital swelling. An incisional biopsy revealed perivascular and periadnexal infiltration of lymphocytes, plasma cells, and a few neutrophils along with pigmentary incontinence. The formation of multiple lymphoid follicles and infiltration by monomorphic lymphoid cells in deeper skeletal muscle fibers were also observed. Immunohistochemistry (IHC) showed polyclonality and low Ki-67 labeling (20%), corresponding to periorbital RLH. Our objective in this study is to highlight the importance of considering PSL as a differential diagnosis in periorbital swelling. We also suggest that recurrent angioedema may lead to PSL.

## Introduction

Pseudolymphoma (PSL) of the orbit is a benign lymphoid hyperplasia (LH). It is a rare disease and must be differentiated from orbital malignant lymphoma [1]. LH is further categorized into "reactive" (RLH) and "atypical" (ALH) based on the presence or absence of unequivocal malignant features. RLH is the most common type of LH. It is now called low-grade B-cell non-hodgkin's lymphoma (NHL) [2]. A recently established clinical entity of LH is IgG4-related disease (IgG4-RD), characterized by fibroinflammatory lesions, high levels of circulating IgG4, and tissue infiltration of IgG4+ plasma cells [3]. Only 25% of cases develop extra orbital lesions. The condition is associated with a five-year mortality rate of 6% [4]. An extensive range of causative agents (e.g., Borrelia infection, injections, tattoos, and arthropod bite) has been described [5]. The most common causes of cutaneous lymphoid infiltrates are drugs such as calcium channel blockers, anticonvulsants, antidepressants, H1 and H2 antagonists, statins, various biologic drugs, and angiotensin-converting enzyme (ACE) inhibitors. Lymphocytoma cutis is a common clinical presentation of drug-induced PSL. It presents as a single or a few plaque-like and/or nodular lesions, particularly on the head, neck, and upper trunk [6]. The diagnostic workup comprises detailed medical history and physical examination, including palpation of lymph nodes [5]. To rule out systemic infectious, lymphoproliferative, and inflammatory disorders, all patients need to get comprehensive infective, hematological, and autoimmune screenings respectively [7]. Tissue biopsy is warranted in all cases as the condition is clinically and radiologically indistinguishable from low-grade malignant lymphoproliferative disorder [2].

The histological diagnosis of RLH is based on the formation of reactive lymphoid follicles of varying size and dense infiltration of small, histologically bland lymphocytes [7]. To rule out the presence of a clonal population, immunohistochemistry (IHC) and/or molecular genetic techniques are used [7]. Some degree of cytologic atypia along with the dense nature of the lymphocytic infiltrate makes the distinction from low-grade lymphoma difficult [5]. Treatment options include the removal of the causative agent; single lesions can be treated by complete surgical excision. Alternative treatment options include cryotherapy and topical or intralesional corticosteroids. In refractory cases, radiation therapy may be considered. In patients with idiopathic multifocal PSL with multiple lesions, systemic corticosteroids or intralesional or systemic interferon alpha or oral hydroxychloroquine can be used. The most important step in preventing the persistence and recurrence of PSL is avoidance of re-exposure to the inducing agent (i.e., vaccines, allergen injections, tattoos, acupuncture, and other drugs) [5]. Intraorbital injection of corticosteroids may have a role as a first-line therapy in RLH of the anterior orbit and may be a useful treatment option for orbital RLH [7]. Rituximab should be considered as another treatment option for orbital PSL [8]. The cutaneous PSL has a variable course and may persist over several months or even years in most cases. In cases induced by drugs or allergens, recurrence can be observed, particularly after re-exposure to the inducing agent [5]. The prognosis is generally favorable for ocular adnexal LH, but the small risk of NHL requires follow-up for at least five years [2]. In this report, we describe a case of periorbital PSL diagnosed in a 58-year-old female who presented with recurrent right periorbital swelling. We aim to emphasize the role of ACE inhibitors in angioedema and highlight the need to consider PSL as a differential in periorbital swelling. We also suggest recurrent angioedema as a possible cause of PSL.

## Case presentation

**Figure 1:**
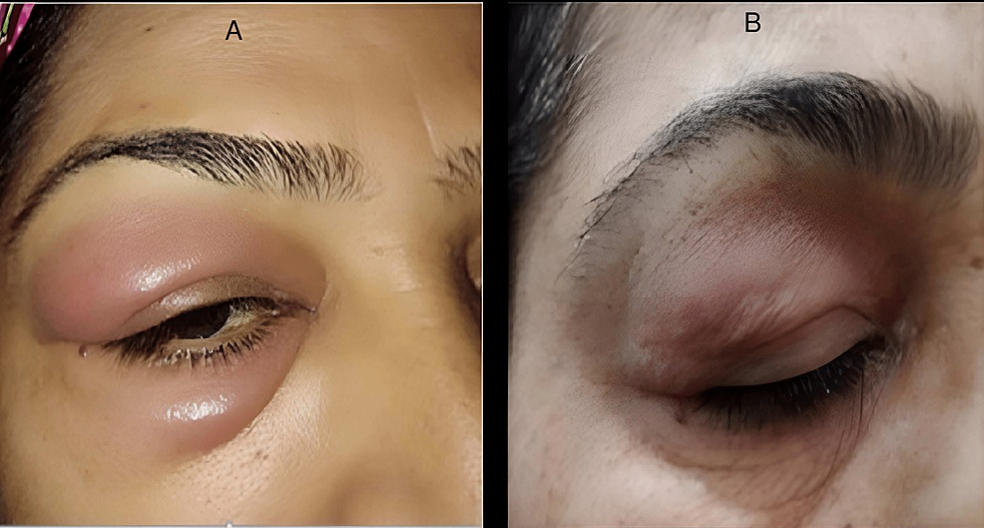
(A) Diffuse right periorbital swelling with mild erythema. (B) The swelling subsided after stopping the ACE inhibitor ACE: angiotensin-converting enzyme

A 58-year-old Pakistani lady, who is a known case of hypertension on ACE inhibitor, presented with an asymptomatic, recurrent right periorbital swelling for three years. The swelling had started at the right lower eyelid and gradually progressed to involve the right upper eyelid over a period of two months. It was associated with mild redness but there was no pain, itching, headache, or fever. There was no history of photosensitivity, night sweats, weight loss, and lumps in the neck, axilla, or inguinal area. There had been no preceding history of recent trauma, vaccination, infection, or acupuncture prior to the appearance of these lesions. On examination, the patient had unilateral, diffuse, right periorbital swelling more prominent on the right upper eyelid as shown in Figure 1A. It was soft, mildly erythematous, non-tender, and with normal local temperature. The rest of the cutaneous, ophthalmological, and systemic examination was unremarkable.

We kept angioedema without wheals and periorbital cellulitis as our top differentials; other differentials that were kept down the list were lymphoma, schwannoma, and orbital IgG4-RD.

Baseline laboratory investigations were within normal range. Other investigations including ANA, ENA profile, RA factor, complement C4 and C1 esterase level, IgE, IgG4, thyroid profile, and fasting blood sugar were also within the normal range. The stool for H. pylori was negative. MRI brain showed left maxillary sinusitis. Based on these findings, most of our differentials were ruled out except for angioedema. The suspected culprit drug in our patient was an ACE inhibitor, which was replaced with a beta-blocker (tab carvedilol). She was also given tab fexofenadine 180 mg along with tab levocetirizine 5 mg, which led to an improvement in her condition, as shown in Figure 1B, and hence we diagnosed her as a case of ACE inhibitor-induced angioedema.

After four months, her right periorbital swelling reoccurred. She was then started on a short course of tab prednisolone 50 mg/day, according to 0.5 mg/kg/day. She received multiple short courses of oral steroids over the next two years. The patient showed a temporary response to oral steroids but swelling reoccurred when the steroid was tapered to 10 mg/day. The patient showed poor response to antihistamines. In addition to steroids, the patient was also given proton pump inhibitors, bisphosphonates, as well as vitamin D and calcium supplements. Ophthalmology and rheumatology opinions were taken. Dietitian and rehabilitation reviews were also taken for dietary modification and muscle-strengthening exercises. She was then started on oral immunosuppressant, i.e., azathioprine 50 mg once daily along with oral steroids for two months but showed no significant improvement. Therefore, the patient was investigated further keeping in mind RLH, low-grade lymphoproliferative disorder, and unicentric Castleman disease.

**Figure 2:**
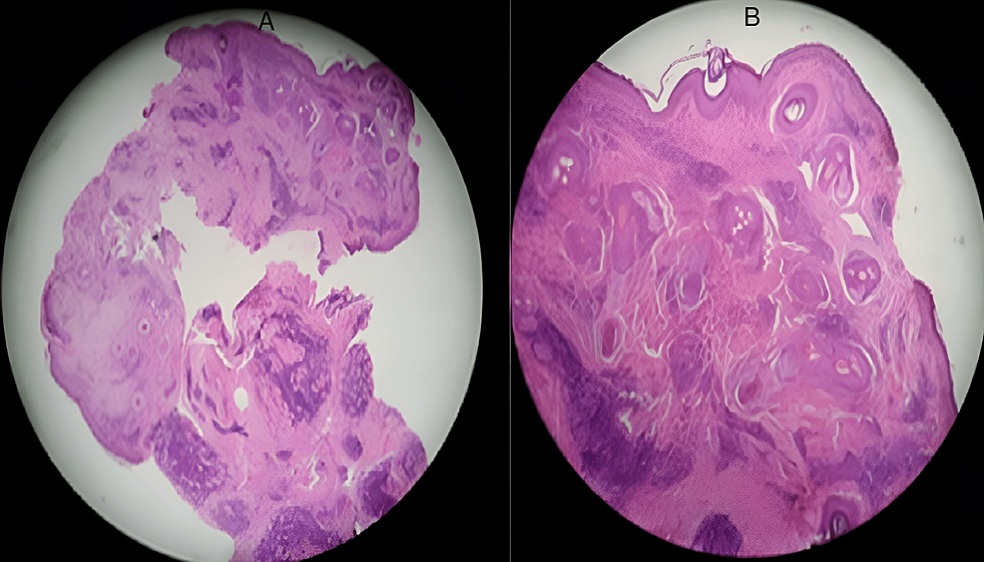
(A) Histopathology showing perivascular and periadnexal mixed cell infiltration, predominantly lymphocytes in the dermis. (B) Formation of multiple lymphoid follicles and infiltration by monomorphic lymphoid cells in deeper skeletal muscle fibers

CT of the head and neck was unremarkable except for right maxillary sinusitis. An incisional biopsy of the right upper eyelid revealed a normal epidermis. Dermis showed perivascular and periadnexal infiltration of lymphocytes, plasma cells, and a few neutrophils along with pigmentary incontinence as shown in Figure 2A. The formation of multiple lymphoid follicles and infiltration by monomorphic lymphoid cells in deeper skeletal muscle fibers were also observed, as shown in Figure 2B. IHC was positive for both B and T cell markers (including CD3, CD20, CD43, and CD5), which showed polyclonality. Ki-67 labeling was low (20%). All these findings were suggestive of periorbital RLH

**Figure 3:**
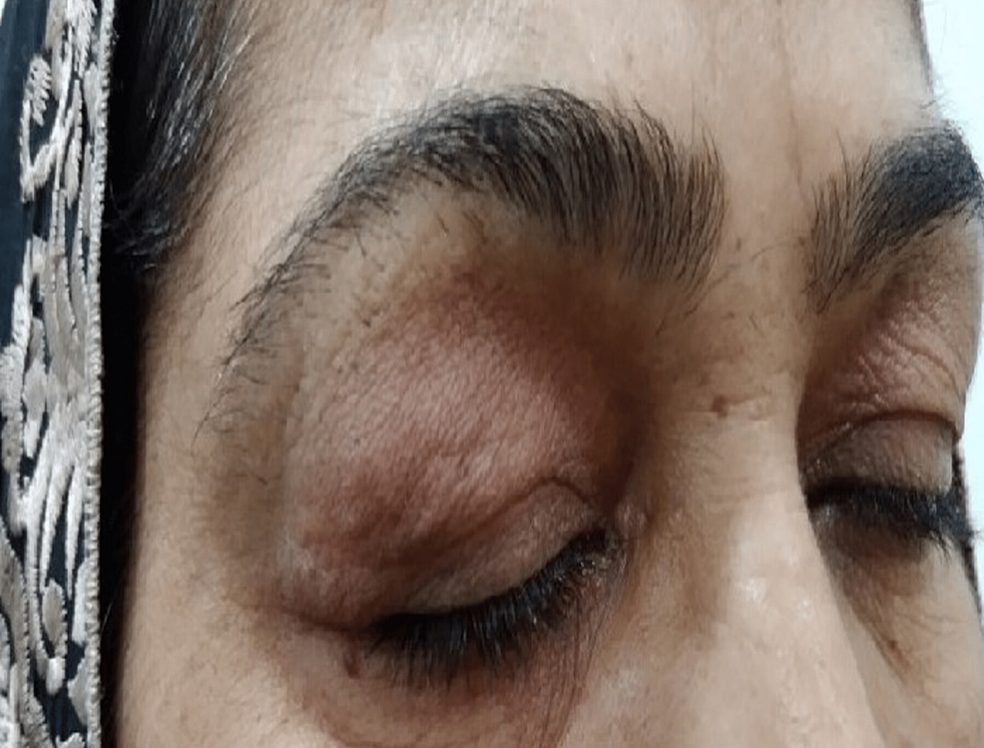
Swelling subsided completely after one course of intralesional steroid

The patient was then referred to an oculoplastic surgeon. She was given an intralesional steroid to which she responded well. The swelling subsided completely, as shown in Figure 3.

She was followed up for six more months, and the swelling did not recur. This patient is still on follow-up for up to five years in order to rule out the small risk of NHL.

## Discussion

RLH and low-grade malignant lymphoproliferative disorders in the orbit and ocular adnexa are clinically and radiologically indistinguishable from each other, requiring tissue biopsy in all cases [2]. Histopathology alone cannot rule out malignancy. Evidence of polyclonality on IHC and low Ki-67 were suggestive of benign nature [9].

NHL may arise concurrently or subsequent to LH in rare cases, necessitating long-term follow-up of patients [2]. Our patient was followed up for six months and there has been no recurrence and no evidence of NHL to date. Hence, we have planned for a longer-term follow-up. Extra orbital lesions develop in 25% of patients [4]. However, there was no extra orbital involvement in our patient. 

The clinical presentation of cutaneous PSL ranges from a solitary nodule and clustered or disseminated papules to erythroderma [6]. In our case, it presented as unilateral, diffuse, mildly erythematous, and painless periorbital swelling. A significant proportion of patients may require repeat intraorbital injections of triamcinolone acetonide to achieve resolution [7]. However, our patient required only a single dose and there has been no recurrence to date.

## Conclusions

ACE inhibitors are a common cause of angioedema, which should be considered in cases of recurring periorbital swelling. We suggest that recurrent angioedema may lead to PSL. Therefore, it should be considered among the causes of PSL. However, data in this regard is scarce and further research should be conducted. We also recommend that non-resolving swelling should be biopsied early to establish a proper diagnosis.

## References

[1] Terada T (2012). Pseudolymphoma of the orbit. Med Oncol.

[2] Andrew NH, Coupland SE, Pirbhai A, Selva D (2016). Lymphoid hyperplasia of the orbit and ocular adnexa: a clinical ​pathologic review. Surv Ophthalmol.

[3] Tokura Y, Yagi H, Yanaguchi H (2014). IgG4-related skin disease. Br J Dermatol.

[4] Jakobiec FA, McLean I, Font RL (1979). Clinicopathologic characteristics of orbital lymphoid hyperplasia. Ophthalmology.

[5] Mitteldorf C, Kempf W (2017). Cutaneous pseudolymphoma. Surg Pathol Clin.

[6] Magro CM, Daniels BH, Crowson AN (2018). Drug induced pseudolymphoma. Semin Diagn Pathol.

[7] Andrew NH, Kearney D, Selva D (2013). Intraorbital corticosteroid injection for orbital reactive lymphoid hyperplasia. Eye (Lond).

[8] Witzig TE, Inwards DJ, Habermann TM (2007). Treatment of benign orbital pseudolymphomas with the monoclonal anti-CD20 antibody rituximab. Mayo Clin Proc.

[9] Terada T (2013). Cutaneous pseudolymphoma: a case report with an immunohistochemical study. Int J Clin Exp Pathol.

